# Exploring biosurfactant from *Halobacterium jilantaiense* as drug against HIV and zika virus: fabrication, characterization, cytosafety property, molecular docking, and molecular dynamics simulation

**DOI:** 10.3389/fbioe.2024.1348365

**Published:** 2024-03-13

**Authors:** Mohammed S. Almuhayawi, Naglaa Elshafey, Nashwa Hagagy, Samy Selim, Soad K. Al Jaouni, Ahmed R. Sofy, Mennatalla Samy, Hattan S. Gattan, Mohammed H. Alruhaili, Mohanned Talal Alharbi, Mohammed K. Nagshabandi, Muyassar K. Tarabulsi, Mohamed E. Elnosary

**Affiliations:** ^1^ Department of Clinical Microbiology and Immunology, Faculty of Medicine, King Abdulaziz University, Jeddah, Saudi Arabia; ^2^ Department of Botany and Microbiology, Faculty of Science, Arish University, Al-Arish, Egypt; ^3^ Department of Biology, College of Science and Arts at Khulis, University of Jeddah, Jeddah, Saudi Arabia; ^4^ Department of Botany and Microbiology, Faculty of Science, Suez Canal University, Ismailia, Egypt; ^5^ Department of Clinical Laboratory Sciences, College of Applied Medical Sciences, Jouf University, Sakaka, Saudi Arabia; ^6^ Department of Hematology/Oncology, Yousef Abdulatif Jameel Scientific Chair of Prophetic Medicine Application, Faculty of Medicine, King Abdulaziz University, Jeddah, Saudi Arabia; ^7^ Department of Botany and Microbiology, Faculty of Science, Al-Azhar University, Nasr City, Egypt; ^8^ Department of Communications and Computers Engineering, The Higher Institute of Engineering, El-Shorouk City, Egypt; ^9^ Department of Medical Laboratory Technology, Faculty of Applied Medical Sciences, King Abdulaziz University, Jeddah, Saudi Arabia; ^10^ Special Infectious Agents Unit, King Fahad Medical Research Center, King AbdulAziz University, Jeddah, Saudi Arabia; ^11^ Department of Medical Microbiology and Parasitology, Faculty of Medicine, University of Jeddah, Jeddah, Saudi Arabia

**Keywords:** biosurfactant, Halobacterium, molecular docking, ADMET, HIV, ZIKA virus

## Abstract

Biosurfactants are surface-active molecules with unique qualities and various uses. Many microorganisms produce secondary metabolites with surface-active characteristics that serve various antiviral functions. The HIV and Zika viruses were chosen for this study because they can spread from mother to child and result in potentially fatal infections in infants. Halophilic bacteria from the Red Sea solar saltern in Egypt were screened using drop collapse, emulsification activity, and oil displacement assays to produce biosurfactants and emulsifiers. *Halobacterium jilantaiense* strain JBS1 was the most effective strain of the Halobacteriaceae family. It had the best oil displacement test and emulsification activity against kerosene and crude oil, respectively. Among the ten isolates, it produced the most promising biosurfactant, also recognized by the GC-MASS library. This study evaluated biosurfactants from halophilic bacteria as potential antiviral drugs. Some of the computer methods we use are molecular docking, ADMET, and molecular dynamics. We use model organisms like the HIV reverse transcriptase (PDB: 5VZ6) and the Zika virus RNA-dependent RNA polymerase (ZV-RdRP). Molecular docking and molecular dynamics make the best complexes with 5VZ6 HIV-RT and flavone (C25) and 5wz3 ZV-RdRP and ethyl cholate (C8). Testing for ADMET toxicity on the complex revealed that it is the safest medicine conceivable. The 5VZ6-C25 and 5wz3-C8 complexes also followed the Lipinski rule. They made five hydrogen bond donors and ten hydrogen bond acceptors with 500 Da MW and a 5:1 octanol/water partition coefficient. Finally, extreme settings require particular adaptations for stability, and extremophile biosurfactants may be more stable.

## 1 Introduction

Extremophilic bacteria proliferate and perform all their metabolic functions in hazardous environments (Deive et al., 2012). In arid environments like the Dead Sea, lakes in Antarctica, and Cuatro Cienegas (Mexico), halophilic bacteria can survive (Marhuenda-Egea et al., 2002; Souza et al., 2006; de Lourdes Moreno et al., 2013; Edbeib et al., 2016). Some of these microbes can tolerate 30% w/v NaCl. Halophilic bacteria are biotechnologically adaptive because they can survive low water and high salinity (Jin et al., 2019). They also produce surface-active chemicals, extracellular and intracellular enzymes, and other molecules (Yin et al., 2015; Barbachano Torres et al., 2020; Elshafey et al., 2022). Amphiphilic biosurfactants are produced by several organisms with hydrophobic and hydrophilic tails (Khemili-Talbi et al., 2015). “Alternative surfactants” fared better than synthetic biosurfactants. Strong biodegradability, tissue selectivity, minimal harm to mammalian cells, minimal irritancy, effectiveness at high or low pH levels, suitability for mass production, and ecological acceptability (Shekhar et al., 2015). Biosurfactants are used in bioremediation of oil-contaminated environments, cosmetics, agriculture, and pharmaceuticals (Ariech and Guechi, 2015). Since extremophiles have unique adaptations to hostile environments, searching for new biosurfactant molecules is promising (Sarafin et al., 2014). Despite the few investigations on biosurfactant-producing species in hypersaline settings, halophilic archaea and bacteria have become more popular for biosurfactant synthesis. Archaea and other halophilic microorganisms can remove and break down complex hydrocarbon molecules in hypersaline environments. This is both cost-effective and good for the environment. In harsh conditions, microorganisms use organic contaminants as their only carbon source to make biosurfactants, which help with bioremediation (Torregrosa-Crespo et al., 2017). Soil, oil fields, seawater, and marine sediments have all been shown to have microorganisms that create biosurfactants. Oil-deposit microorganisms are better able to survive in harsh environments than those of other oil resources. Oil ponds are home to species of *Bacillus*, *Acinetobacter, Rhodococcus, Enterobacter, Dietzia, Paenibacillus*, and *Pseudomonas*. These species all make biosurfactants, which are things like lipopeptides, glycolipids, phospholipids, and fatty acids. Amphiphilic lipopeptides, according to (Kuyukina et al., 2016), have high surface activity. *Bacillus* species lipopeptidesBacillus species, such as B. licheniformis, *B. subtilis*, B. cereus, B. liquefacient, and Halomonas sp make lipopeptides. BS4 (Donio et al., 2013) generates a biosurfactant with biomedical significance identified from solar salt operations in India. *Salibacterium* sp. was discovered recently (Villa-Rodriguez et al., 2018). Microbial surfactants (MS) contain various compounds, including peptides, fatty acids, phospholipids, glycolipids, antibiotics, and lipopeptides. Most biosurfactants are good biomolecules for making specialty chemicals, biological control agents, and new-generation compounds for the healthcare, cosmetics, and pharmaceutical industries because they can kill bacteria, fungi, or viruses. (Kuyukina et al., 2016). Conversely, viruses have been responsible for pandemics and significant epidemics worldwide (Yeh et al., 2019), on the other hand, lipid bilayer around virion and capsid of envelope viruses has viral proteins that help the virus stick to host cells. The genetic makeup of non-envelope viruses is housed within the protein layer of the capsid. The two primary routes of transmission for envelope viruses are respiratory transmission (e.g., SARS-CoV2) and body fluid transmission (e.g., hepatitis viruses, herpes simplex virus (HSV), and human immunodeficiency virus type 1). Envelope viruses, believed to be the cause of pandemics and other significant disease outbreaks, pose a serious threat to human health (Silverman and Boehm, 2021). Human immunodeficiency virus infection is widespread worldwide (De Cock et al., 2021). When the immune system is substantially impaired, HIV infection progresses to AIDS (Carriero et al., 2020). According to (Simon et al., 2006), HIV causes a progressive immune system problem, making the body more susceptible to infections that might cause secondary neoplastic illnesses and respiratory, cardiovascular, and neurological dysfunctions. In an infected human, these viruses can co-exist and serve as co-factors to enhance disease morbidity and mortality. To enhance the detection of these viral infections by employing, susceptible diagnostic tests, it is advantageous to possess thoroughly defined virus samples that may be used to adjust and confirm the tests. Similarly, the availability of large-scale, high-titer virus preparations can considerably boost efforts toward disease prevention through vaccine development. Because of this, it is important to use good methods for growing large cultures so that it is easier to make diagnostic evaluation panels and formulate possible vaccines and new medicines (Manak et al., 2012). The conventional techniques for cultivating these viruses in a laboratory setting involve virus isolation and the utilization of mammalian cell co-cultures, which are conducted in Bio-Safety Level 2 or 3 (BSL-2 or 3) facilities. These procedures incur expenses associated with consumables for monitoring cultures (Manak et al., 2012). The HIV protein reverse transcriptase (RT) is a promising therapeutic target because it converts the ssRNA viral genome into the dsDNA provirus. A different virus-coded protein imports and integrates the provirus into the host genome. In the last year, the Zika virus (ZIKV) has raised worries about public health (Simon et al., 2006). According to Carteaux and Driggers (Driggers et al., 2016; Elfiky, 2016), Infection with ZIKV during pregnancy has been linked to microcephaly in both the fetus and the newborn. Antiviral drugs work by disrupting the viral replication machinery. In the cytoplasm of infected cells, the RNA genome of ZIKV self-assembles into a polyprotein. Host or viral proteases. The polyprotein is processed into three structural and seven nonstructural (NS) proteins. Viral particles are composed of structural proteins called the precursor membrane (prM), the envelope (E), and the capsid (C). Proteins NS1, NS2A, NS2B, NS3, NS4A, NS4B, and NS5 affect cells, polyprotein processing, and genome replication during viral infections. Host cofactors and NS proteins come together on ER membranes during RNA replication to form a multi-protein replication complex (RC) (Elnosary et al., 2022). The most significant and most conserved RC protein is one of them, NS5. Two key NS5 enzymatic activities, methyltransferase (MTase) and RNA-dependent RNA polymerase (RdRp) are potential targets for creating antiviral drugs since they are necessary for viral replication. On the other hand, Workers in laboratories worldwide face the potential danger of contracting viral infections while handling newly discovered viruses. The rising number of virological research activities involving Risk Group 3 or 4 (Pedrosa and Cardoso, 2011) has led to a growing concern about accidental viral infections among, hospital workers, or research facilities. Although the precise risk of infection following virus exposure is not yet known, bloodborne emerging viruses like hepatitis C and HIV are the most frequently found viral infections (Singh, 2011). Additionally, there have been reports of laboratory-acquired infections due to other new viruses like SARS, Marburg, dengue, vaccinia, Crimean-Congo hemorrhagic fever, Western equine encephalitis, Ebola, West Nile virus, and Zika viruses (Wong et al., 2016). In a laboratory setting, the primary modes of infection are inhalation, ingestion, contact with mucosal membranes, self-inoculation, and direct contact with animal or insect vectors (Sewell, 1995). To develop new therapeutic medications with an enhanced efficacy and safety profile for these diseases, it is critical to keep searching for novel anti-HIV and anti-Zika virus agents (Perfumo et al., 2018; Elnosary et al., 2023). Biosurfactants, or surfactants generated from microorganisms, have gained popularity as a natural means of preventing tropical diseases. These biosurfactants are intriguing materials for biological applications because of their low toxicity, pH stability, biodegradability, thermal resistance, and biological activity potential. When synthetic equivalents are used, their biological effects can’t be found. The principles of viral illness (Santos et al., 2016; Sajid et al., 2020; Sofy et al., 2020; ZeinEldin et al., 2023) and biosurfactants (production, extraction, characterization, and confirmation assays). Biosurfactant-inactivated envelope viruses are better than non-envelope viruses. The physical and chemical properties of biosurfactants hurt the lipid membranes of envelope viruses by reacting with the hydrophobic domain (Sofy et al., 2020). Researchers are seeking safe and effective broad-spectrum antivirals. Viral chemoresistance makes direct virucidal. Biosurfactant impact is crucial to developing alternative medicinal agents. Biosurfactant also has immunomodulatory and anti-inflammatory properties (Jain et al., 1991; Bodour and Miller-Maier, 1998). Thus, this study investigates a promising *Halobacterium jilantaiense* strain JBS1 of biosurfactant active components as a therapy for HIV and Zika viruses. Current work uses molecular docking, ADMET, and molecular dynamics.

## 2 Materials and methods

### 2.1 Isolation and identification of halophilic bacteria

Sediment samples were taken from the Egyptian Red Sea Solar Saltern. The samples were taken in clean polythene bags and kept in refrigerators at a temperature of 4°C. On the first collection day, a microbiological examination was conducted after arrival at the lap. The sterile nutrient agar pour-plates method used 1 mL of each gram of serially diluted soil sample and 20% NaCl. The plates were incubated at 37°C for 7 days.

### 2.2 Screening of potential halophilic biosurfactant-producing bacteria

SevenTen halophilic bacterial isolates were collected after 7 days at 37°C. The cultivated broth’s centrifuged supernatants were examined using a variety of screening techniques to identify the most probable biosurfactant producers as follows.

#### 2.2.1 Drop collapsing test

2 µL of mineral oil was applied to the wells of a microtiter plate. 5 µL of the culture supernatant was added to the oil’s surface after the lid had been at room temperature for an hour to equilibrate. The drop’s shape on the oil surface was evaluated after a minute. Cultures that produced biosurfactant and flat droplets received positive “+” scores. Following research Maneerat and Phetrong (2007), Selim et al. (2012), Mouafi et al. (2016), round drops from cultures were graded negatively, indicating that no biosurfactant was being created.

#### 2.2.2 Oil displacement test

The Petri dish was filled with 50 mL of distilled water, and then 200 μL of crude oil was added to the water’s surface. Ten μL of cell-free culture broth was added to the top of the crude oil.The diameter of the clear zone on the oil’s surface was measured and compared to 10 μL of distilled water (Bulmus, 2003).

#### 2.2.3 Emulsification activity

To assess emulsification activity, we used a modified version of the Cooper and Goldenberg method from 1987. In a nutshell, 4 mL of kerosene and 4 mL of culture supernatant were combined for 5 min and then vortexed quickly. The mixture stood for 10 min before measurement. Emulsification activity, according to (Kuyukina et al., 2016), is calculated as a percentage by dividing the height of the emulsion layer by the overall height. To calculate the E24, divide the liquid height of the displayed liquid emulsion layer by the displayed liquid’s height.

The equation for E24 (%): (h emulsion/h total) ×100, where h emulsion is the height of the emulsion layer, h total is the height of the liquid, and E24 is the emulsion index after 24 h. The potential strain was selected, and additional investigations employing the potential bacteria were done based on the positive findings from these screening techniques.

### 2.3 Hemolytic assay

The hemolytic assay was conducted using the methodology described in Bulmus (2003). The sheep red blood cells were promptly collected and subjected to a triple washing procedure using a 150 mM NaCl solution at a centrifugation speed of 2,500 g for 10 min. The plasma was extracted, and the cells were subsequently immersed in a phosphate-buffered saline solution with a pH of 7.4, resulting in a concentration of 2% red blood cells. A series of double-folded dilutions with concentrations of 1000, 800, 600, 400, 200, 100, and 50 μg/mL were prepared using an extract. Each dilution was combined with 2% L of red blood cell (RBC) solutions. The final reaction mixture volume was adjusted to 1 mL by adding pps. Subsequently, the reaction mixture was subjected to a temperature-controlled water bath set at 37°C for 1 h. Following the designated incubation period, the reaction mixture underwent a subsequent centrifugation step at a speed of 2,500 g for 15 min. The supernatant was collected, and the optical density was measured at a wavelength of 541 nm, with phosphate-buffered saline used as the blank. The positive control in this study involved the use of deionized water. The experiment was conducted in triplicate, and the mean ± standard deviation (SD.) was computed.

Percentage hemolysis = (Absorbance of sample- Absorbance of blank) × 100/Absorbance of positive control.

### 2.4 Molecular identification of the potential bacterium

Using a method described by Maloy (Maloy, 1990) in Experimental Techniques in Bacterial Genetics, genomic DNA was extracted from bacterial strains. The universal bacterial primer (Invitrogen, United States) was used to amplify genomic DNA (100 ng) from the most potent halophilic bacterium. According to (García et al., 2004), two such sequences are 27F (5′-AGAG TTTG ATCM TGGC TCAG -3′) and 1492R (5′-GGTT ACCT TGTT ACGA CTT -3′). These are the parameters for the PCR: We ran 30 cycles with 50 L of the reaction system at 95°C for 5 min before denaturation, 94°C for 1 min of denaturation, 60°C for 1 min of annealing, 72°C for 1 min and 30 s, 72°C for 10 min, and 4°C for holding. Following the protocol provided by the MacroGen Company of Korea (http://www.macrogen.com), samples containing 50 ng/L of each PCR product were made and shipped to the MacroGen Lab. The strains were predicted using BLAST (http://www.ncbi.nlm.nih.gov/BLAST/) analysis of the sequences. Cluster analysis was performed using MEGA 11 to check the probable bacteria’s 16S rRNA gene sequences against the GenBank database of other bacterial sequences.

### 2.5 Extraction of biosurfactant

Adsorption chromatography was used to clean acid precipitates repeatedly, which let the biosurfactant be taken out of the cell-free broth where cells had been growing for 72 h. To remove the surfactant from the bacterial media, we centrifuged it at 10,000 g for 20 min. An aliquot of 6 N HCl was added to the supernatant to lower the pH to 2.0, and the residue was then allowed to develop at 4°C. The precipitate was pelleted for 20 min at 10,000 g, then freeze-dried, the pH was corrected, and the residue was redissolved in clean water and weighed. After acetone extraction (Deka and Das, 2009), the surfactant was vacuum-dried using a rotary evaporator.

### 2.6 GC-MASS analysis of biosurfactant

A Trace GC1310-ISQ mass spectrometer (Thermo Scientific, Austin, TX, United States) set up with a direct capillary column TG-5MS (30 m × 0.25 mm x 0.25 m film thickness) was used to determine the chemicals in the samples. After 2 minutes, the column oven’s temperature was raised from 50°C to 230°C per minute. Helium was used as the carrier gas, and temperatures in the injector and MS transfer line were steady at 250°C and 260°C. To determine which components were present, we compared mass spectra and retention durations to the databases at WILEY 09 and NIST 11.

### 2.7 Molecular docking

#### 2.7.1 Receptor preparation

We downloaded the crystal structures of ZIKV NS5 RNA-dependent RNA polymerase (ZV-RdRP) and HIV reverse transcriptase (HIV-RT) from the protein data bank (PDB) database of the Research Collaboration for Structural Bioinformatics (RCSB) (Berman et al., 2000). To facilitate the creation of hydrogen bonds between the ligand and the target, water (solvent) molecules were incorporated into the conserved active area of the receptor protein using the MGL-tools program. The document was then pdpqt-formatted for safekeeping.

#### 2.7.2 Ligands preparation and optimization

Twenty-six ligands were designed in ChemDraw Professional 15.0 for this study. The ligands’ 3D structures were built and saved as SDF files using Open Babel (O Boyle et al., 2011) for later processing and molecular docking studies.

#### 2.7.3 Molecular docking

The auto-dock_vina_1_1_2_linux_x86 program was utilized to run the molecular docking and scoring computations. Crystallographic information at a resolution of 2.7 A was obtained for HIV-RT (PDB ID: 5VZ6). The crystal structure of ZV-RdRP (PDB ID: 5wz3) was determined at a resolution of 1.8. Rigid receptors and ligands were used in the docking simulation. The grid boxes and three-dimensional structures of HIV-RT and ZV-RdRP may be found in Supplementary Table S1. The HIV-RT structure is a 5VZ6, and the ZV-RdRP structure is a 5wz3. On the other hand, the visualization was done using BIOVIA Discovery Studio 2021.

### 2.8 Predicted pharmacokinetic and toxicity properties

Drug kinetics is relevant in this context. ADMET is an acronym that represents the processes of absorption, distribution, metabolism, and excretion. [A] Absorption describes how a medicine enters the bloodstream from the delivery site. [D] Distribution is the term used to describe blood movement into and out of tissues. [M] Enzymes frequently carry out metabolism, which is the chemical transformation of the medication to discard it. The parent drug is completely and permanently removed from the body through elimination. [T] Toxicology This filter, as its name suggests, evaluates the significance of a chemical and its metabolites. (Burton et al., 2021; Rbaa et al., 2021). Pfizer’s Lipinski developed a set of guidelines for writers of easily accessible chemicals. The four suggestions are: (Deive et al., 2012): The compound’s molecular weight (MW) must be 500 g/mol or below. (de Lourdes Moreno et al., 2013). The molecule should only include five hydrogen bond donors, most of which are OH and NH groupings. (Edbeib et al., 2016). Each molecule should not have more than 10 hydrogen bond acceptors. (Marhuenda-Egea et al., 2002). These are the oxygen and nitrogen atoms of the molecule, respectively. The molecule has a maximum logP-measured lipophilicity of 5. The term “Lipinski’s Rules” or “Rule of 5” has been used to describe these (Protti et al., 2021). To have a reasonable possibility of having favorable oral bioavailability, the molecules should follow at least three of the four parameters, according to Chen et al., 2020 Lipinski’s research. Therefore, when we think about absorption, efficacy, and toxicity (ADME), Lipinski’s principles help us meet the letter A. Solubility and membrane permeability are the two most important factors in oral absorption, and these suggestions focus only on them. Toxicological risks can be mitigated with the help of computational methods, such as the Simplified Molecular Input Line System’s (SMILES) use of the SwissADME program (Hajji et al., 2021).

### 2.9 Molecular dynamics simulations

A molecular dynamics simulation of the protein-ligand complexes was performed using the Linux 5.4 package and GROMACS, version 2021.1. The most active compound, proanthocyanidin-B2, was docked to the structures of the zika virus reverse transcriptase protein (PDB ID: 5wz3) and the human immunodeficiency virus reverse transcriptase protein (5VZ6). The MD simulations were run using the default parameters in GROningen MAchine for Chemical Simulations (GROMACS) version 5.1.2. The PRODRG server (Schüttelkopf and Van Aalten, 2004) and the “pdb2gmx” program (to generate ligand topologies) were used to generate protein topologies. To solve the complexes, the GROMOS96 54a7 force field was used to find their energy-minimal conformations, and then the single point charge (SPC) water model was applied to the resulting rectangular box with 1.0 nm padding. The “gmx genion” script was used to standardize the systems’ net charges. The energy of the complexes was minimized using a force of 10.0 kJ/mol and a maximum of 50,000 steps using the steepest descent minimization approach. The systems were stabilized by employing ensembles of particles, volumes, and temperatures known as NVT and NPT, respectively. Both ensembles were operated at 300 K and 1 atm for 100 ps. For our model, we decided on the Parrinello-Rahman barostat and the V-rescale thermostat. The production run was completed at 300 K with an average time step greater than 100 ns, or 2 fs. For a more in-depth analysis of the complexes’ stability, we calculated their radius of gyration (Rg), solvent accessible surface area (SASA), number of hydrogen bonds, root mean square deviation (RMSD), and root mean square fluctuation (RMSF). The GRACE software was used to make the images. We ran one final production cycle after computing the MM/PBSA binding free energies with the g_mmpbsa GROMACS method. To determine the binding energies, this approach uses the following equation:

$$\Delta \text{Gbinding}=\text{Gcomplex}‐\;\left(\text{Gprotein}+\text{Gligand}\right)$$

where ΔGbinding is the overall binding energy of the complex, Gprotein is the free protein binding energy, and Gligand is the unbounded ligand binding energy.

## 3 Results

### 3.1 Identification of the potential halophilic strain

**FIGURE 1:**
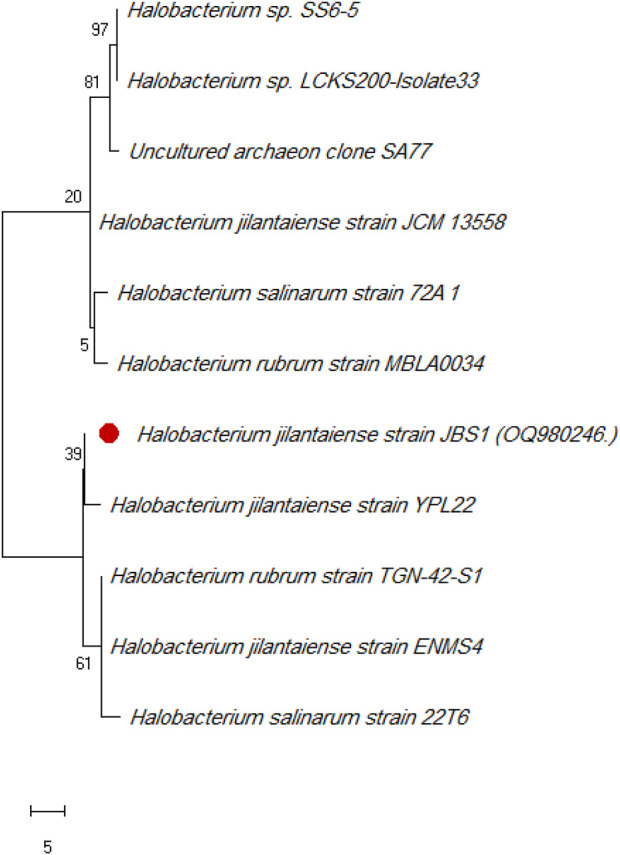
Phylogenetic tree of *Halobacterium jilantaiense* strain JBS1. The Maximum Like-lihood method and General Time Reversible model are used to illustrate the percentage of trees in which the related taxa clustered together next to the branches. The scale of the tree equals the number of substitutions per site divided by the branch lengths. The evolutionary studies were conducted using MEGA 11 software.

*Halobacterium jilantaiense* strain JBS1 was identified as the promising biosurfactant-producing halophilic bacteria among the ten Red Sea solar salt-ern isolates by morphological and 16S rRNA sequencing. Then uploaded it to GenBank with the accession number (OQ980246). The *H. jilantaiense* strain JBS1 is gram-negative, rod-shaped, exhibits optimal growth at 3M NaCl and pH 7, and shares more than 98% of its 16S rRNA sequence with other species, according to phylogenetic and evolutionary research (Figure 1).

### 3.2 Isolation and hemolytic assay of biosurfactant

The *H. jilantaiense* strain JBS1 produced biosurfactants in culture broth after 7 days at 37°C. The cultivated broth’s supernatants were centrifuged to separate the biosurfactants in pellet form, Supplementary Figure S1A

The observations recorded as percentage hemolysis due to biosurfactant treatment are presented in (Table 1). It indicates that biosurfactant concentrations ranging from 200 ug/gl to 1000 cause 0.1% and 0.6% hemolysis, respectively. While 100% hemolysis was observed in deionized water used as a positive control, no hemolysis was observed with a concentration of 100 ug/gl.

**TABLE 1:** Cytosafety property of biosurfactant isolated from *Halobacterium jilantaiense* strain JBS1.

Sample (Conc. Ug/ml)	Mean of sample with RBCs	Sample with isotonic solution Ab	Hemolysis %
Control Complete Hemolysis	1.693 ± 0.002	0.002	100
1000	0.026 ± 0.004	0.015	0.6
800	0.016 ± 0.003	0.01	0.4
600	0.009 ± 0.002	0.006	0.2
400	0.004 ± 0.001	0.002	0.1
200	0.002 ± 0.001	0.001	0.1
100	0.001 ± 0.001	0.001	0.0
0	0.000 ± 0.001	0	0.0

### 3.3 Biosurfactant screening

*Halobacterium jilantaiense* strain JBS1 was the most potential strain able to produce biosurfactants and highly positive in various biosurfactant screening assays, such as Oil displacement and emulsification activity, which are depicted in Supplementary Figures S1A, B.

### 3.4 Chemical analysis of biosurfactant by GC-MASS

The study of bioactive phytocompounds in biosurfactants involved GC-MS analysis, revealing 26 compounds with diverse chemical structures and potential biological activities (Supplementary Table S2 and Supplementary Figure S2). These compounds include 1,2,3-propanetriol, 1,2-diacetate, Triacetin, 1H-Cycloprop [e]azulen-7-ol,decahydro-1,1,7-trimethyl-4-methyle, Ent-Spathulenol, Pentadecanoic acid, Oxirane undecanoic acid, Hexadecanoic acid, Ethyl iso allocholate, Tetraneurin A, 1-Heptatriacontanol, Octadecadienoic acid, Ethyl linoleate, Isopropyl linoleate, Tricyclo [20.8.0.0 (7,16)] triacontane, 1(22),7 (16)-diepoxy-, E,E,Z-1,3,12-Nonadecatriene-5,14-dIol, Stigmast-5-en-3-ol, 10,13-DIOXATRICYCLO [7.3.1.0 (4,9)] tridecan-5-ol-2-carboxylic acid, 4-methyl-11-(1-propenyl)-, methyl ester, Diisooctyl phthalate, 1,2-benzenedicarboxylic acid, Phthalic acid, Trilinolein, 4H-1-benzopyran-4-one, 2-(3,4-dihydroxyphenyl)-6,8-Di-á-d-glucopyranosyl-5,7-dihydroxy-, Flavone, and Rhodopin,. The Flavone class compounds have been studied for their potential antiviral and antimicrobial properties. Likewise, apigenin, and luteolin have shown antimicrobial effects against bacteria, fungi, and viruses by disrupting viral envelopes, inhibiting replication, and interfering with viral entry into host cells. Pentadecanoic acid, an saturated fatty acid found in various plant and animal oils, has diverse biological activities, including in the diet to support long-term metabolic and heart health. Further, Oxirane undecanoic acid, an unsaturated fatty acid found in biosurfactant, possesses diverse biological activities, including anti-inflammatory and antioxidant properties. However, its specific antiviral activity and mechanism are not well-documented. In addition, several peaks in the GC-MS chromatogram complement the findings of phthalic acid, which accounted for most of the twenty-six compounds and 36.73% of all the ingredients. Extensive study has been carried out to investigate the antiviral characteristics of this substance, with a particular focus on its effectiveness against lipid-enveloped viruses including Zika virus and HIV. 3-(pyrimidin-2-yl)-N-[3-(5,6,7,8-tetrahydronaphthalen-2-yl)-1H-pyrazol-5-yl] propenamide10,13-dioxatricyclo [7.3.1.0 (4,9)] tridecan-5-ol-2-carboxylic acid, 4-methyl-11-(1-propenyl)-, methyl ester, and CIS-2-phenyl-1,3-dioxolane-4-methyloctadec-9, 12, 15-trienoate, are all compounds with specific antiviral activities and mechanisms. Importantly, the study of bioactive phytocompounds in biosurfactants provides valuable insights into its potential health benefits and potential antiviral properties.

### 3.5 Molecular docking

To find compounds with therapeutic action against the HIV and Zika viruses, three molecules obtained from halophilic bacteria were docked using the autodock_vina_1_1_2_linux_x86 software. It is a method that enables the analysis or prediction of the interactions that are the primary determinants of a ligand’s affinity for a receptor. Additionally, this research aids in selecting potent ingredients for biosurfactant extract, a substance thought to be a cure for the HIV and Zika viruses. Supplementary Table S2 includes a list of the 26 biosurfactant chemicals as well as two proteins that are well-resolved and available in the Protein Data Bank (ID PDB: 5VZ6 for HIV-RT and 5wz3 for ZV-RdRP (Supplementary Figure S3). According to molecular docking studies, the most significant total score strongly indicates a substantial binding affinity. The biosurfactant compound’s molecular docking binding affinity score ranged from −5.5–13 kcal/mol against the HIV target and from −5.6–10.1 kcal/mol against the Zika virus target. The best complex is [HIV-RT- C25] because it has low energy with a value of 13 kcal/mol, followed by [HIV-RT- C3] with a value of 11.8 kcal/mol compared to the others with a value of 13 kcal/mol, and then [HIV-RT- C24] with a value of 10.7 kcal/mol compared to the reference ligand which has low energy Additionally, [ZV-RdRP-C24] is the most effective complex against the Zika virus target due to its low energy level of—10.1 kcal/mol has an energy value of 10 kcal/mol, which is lower than the other compounds’ values of 13 kcal/mol, and [ZV-RdRP-C8]. has minimal energy with a value of 9.6 kcal/mol, whereas the reference ligand has a value of 9.8 kcal/mol (Table 2).

**TABLE 2:** Molecular docking Binding affinity score of biosurfactant molecules against HIV-RT and ZV-RdRP.

Compound code	Binding affinity score kcal/mol	
	HIV-RT	ZV-RdRP
C1	−5.5	−6.2
C2	−6.4	−6.4
C3	−11.8*	−8.3
C4	−8.1	−7.9
C5	−6.8	−6.1
C6	−8.4	−6.6
C7	−6.4	−6
C8	−8.6	−10.8*
C9	−8.4	−9.2
C10	−7.2	−5.6
C11	−5.7	−6.7
C12	−8.7	−6.4
C13	−7.4	−5.9
C14	−6.6	−5.7
C15	−9.9	−10*
C16	−9.3	−6.5
C17	−9.5	−9
C18	−7.9	−9
C19	−8.8	−7.2
C20	−6.1	−5.4
C21	−7.1	−7.2
C22	−7.2	−7.2
C23	−6.4	−6
C24	−10.7*	−10.1*
C25	−13*	−8.7
C26	−10	−8.2
Ref. ligand	−10.7**	−9.6**

**FIGURE 2:**
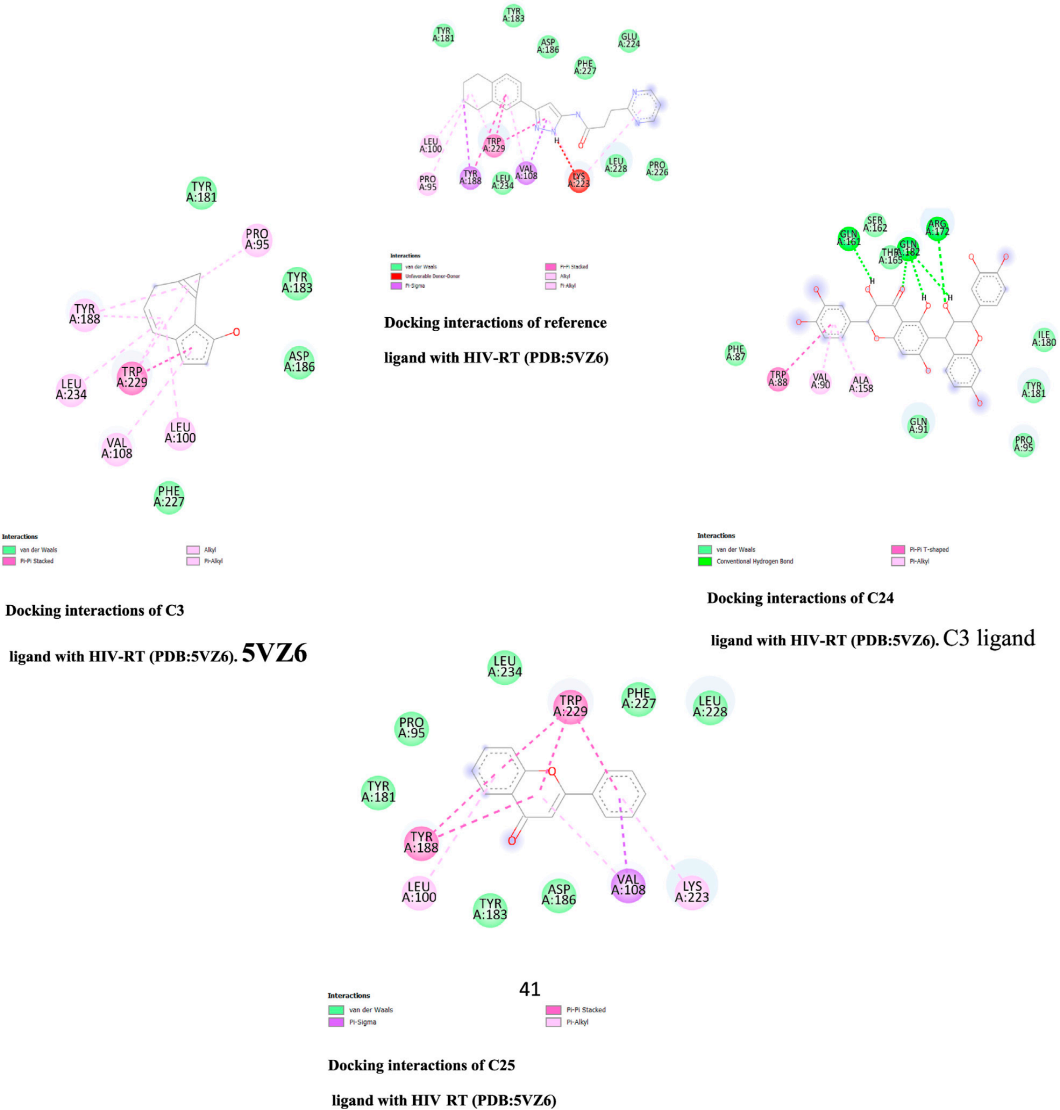
Docking interactions three top ranked molecules and reference ligand with HIV-RT.

**FIGURE 3:**
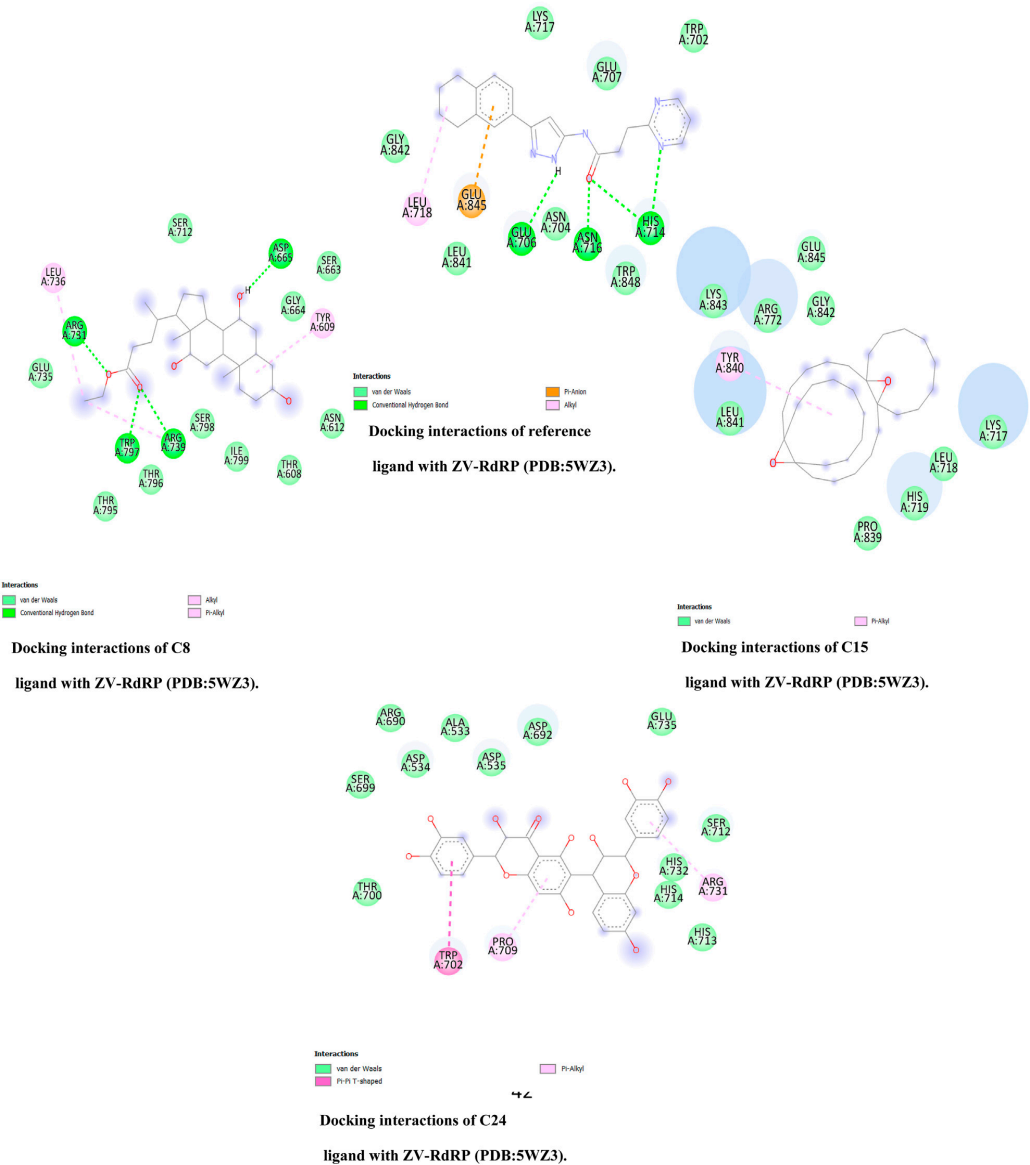
Docking interactions of three top-ranked molecules and reference ligands with HIV-RT.

There are only van der Wall contacts with the residues TYR 181, TYR 183, ASP 186, and PHE 227 for PDB HIV-RT (PDB ID: 5VZ6) (2.7) (Figure 2), whereas Pi-Alkyl-type interactions are detected for C3 molecules with the residues PRO 95, LEU 100, VAL 108, LEU 234, and TYR 184. However, when C24 molecules docked with the same protein are seen, the protein’s binding modes are highlighted in green. These amino acid residues include LYS 717, LEU 718, HIS 719, ARG 792, PRO 832, GLY 842, LYS 843, and GLU 845. Additionally, this combination offers additional Pi-Alkyl interaction with TYR 840 residue. In addition, C25 molecules docked with PDB ID: 5VZ6 provided the best protein binding modes and displayed van der Waals interactions with the residues PRO 95, TYR 181, TYR 183, ASP 186, PHE 228, LEU 228 and LEU 234, Pi-Alkyl with LEU 100, and LYS 223. Additionally, the ZV-RdRP complex (PDB ID: 5wz3; Resolution: 1.8) (Figure 3) with C8 molecules results in hydrogen bonds with the OH groups, demonstrating the significance of the amino acids ASP 665, ARG 731, ARG 739, and TRP 797, which are among the crucial components of protein-ligand stability. On the other hand, when C15 molecules are seen docked with the same protein, the binding modes of the protein are highlighted in green for amino acid residues that interact with GLN 161, ARG 172, and GLN 182. Additionally, this compound interacts with the residues VAL 90 and ALA 158 with a Pi-Alkyl interaction. Additionally, docked C24 molecules with the same protein revealed van der Waals interactions with residues ALA533, ASP 534, ASP 535, ARG 690, etc., and Pi-Alkyl with PRO 709 and ARG 731.

### 3.6 Validation of the docking technique

It is essential to verify the molecular docking process. Using the re-docking method, which forms a new complex (docked), the reference ligand and target protein were downloaded to the PYMOL program in this investigation. When docked with HIV-RT (PDB ID: 5VZ6), the reference ligand has the lowest energy of −10.7 kcal/mol and van der Waals interactions with residues TYR 181, TYR 183, ASP 186, PHE 227, GLU 224, PRO 226; LEU 228; and LEU 234. This illustrates how precisely the docking results match the crystallographic results.

### 3.7 ADMET and Lipinski’s rules

Novel compounds must first be examined for pharmacokinetic characteristics before they can be classified as potential medicines. Therefore, it is believed that using online services offered by Pkcsm. The best way to determine and select the target molecules is by studying ADMET properties.

#### 3.7.1 Absorption

According to a literature survey, drugs or chemicals that produce results (>30% abs) penetrate the intestinal barrier more successfully. The findings demonstrate that the highly ranked compounds in the study had high values; oral absorption in people ranged from 79.29% to 97.70%, demonstrating that these substances were efficiently absorbed.

#### 3.7.2 Distribution

The blood-brain barrier permeability values for HIV-RT distribution suggested that the biosurfactant molecule of interest, C25, had a low value of −1.7. With a low ZV-RdRP score of −0.80, the Blood-Brain Barrier Permeability of C8 shows that it does not cross into the brain. There will be less side effects due to their potency and drug-like properties (Elamin et al., 2017; Almuhayawi et al., 2023a).

#### 3.7.3 Metabolism

Computational metabolic behavior is a biochemical indicator of pharmaceuticals in the body; CYP is crucial for converting drug compounds. The two most important CYP subtypes, 2D6 and 3A4, are substrates and inhibitors of C8 and C25, as indicated in Table 3 (Norinder and Bergström, 2006; Deka and Das, 2009; Al-Mokadem et al., 2022; Almuhayawi et al., 2023b). They might be able to metabolize in the liver, according to this indication.

**TABLE 3:** The results of the ADMET test with pKCSM of top-ranked and ref. compounds docked against HIV-RT and ZV-RdRP.

Compounds	Absorption	Distribution		Metabolism CYP			
	Intestinal absorption (human)	Blood-brain barrier permeability	CNs permeability	2D6 substrate	3A4 substrate	2D6 inhibitor	3A4 inhibitor
	Numeric (l % absorbed)	Numeric (log BB)	Numer-ic (log PS)	Categorical (Yes/No)			
C3	93.18	0.643	−1.99	NO	NO	NO	NO
C8	97.70	−0.80	−2.26	NO	NO	NO	NO
C15	91.30	1.47	−4.39	NO	NO	NO	NO
C24	79.29	1.70	−3.754	NO	NO	NO	NO
C25	97.39	−1.8	−1.33	NO	NO	NO	NO
Reference ligand	94.73	−1.33	−2.61	NO	Yes	NO	Yes

Compounds	Excretion	Toxicity	
	Renal OCT substrate	AMES toxicity	Hepatotoxicity
	Categorical (Yes/No)		
C3	NO	NO	NO
C8	NO	NO	NO
C15	NO	NO	NO
C24	NO	NO	NO
C25	NO	NO	NO
Reference ligand	NO	NO	Yes

#### 3.7.4 Toxicology and waste disposal

C8 and C25 have been demonstrated to be non-toxic in both the AMES toxicity test and the hepatotoxicity test, proving their efficacy, and their excretion results are a sufficient negative value.

#### 3.7.5 Lipinski’s rule

The ADMET technique showed that the biosurfactant molecule of interest to us, C24, was the safest and least toxic of all possible candidates. Additionally, as shown in Table 4, these three compounds meet the criteria for Lipinski’s rule: they have a MW of 500 Da, a 5 octanol/water partition coefficient, and generate 5 H-bond donors and 10 H-bond acceptors. Lipinski’s criteria and the ADMET study were used to digitally validate the produced C8 & C25 as a safe pharmaceutical compound. In line with Lipinski’s principles, the compounds had drug-like features (Table 4), which predicted well for their oral bioavailability. These chemicals met Lipinski’s criterion for psychoactive drugs.

**TABLE 4:** Lipinski’s parameters of all three top compounds and reference in the dataset.

Compounds	Lipinski rule					
	HBD	HBA	MW	Log P	t-PSA	RB
C3	1	1	156.18	2.4	20.23	0
C8	3	5	436.63	3.93	187.94	5
C15	0	2	444.74	9.19	198.52	0
C24	9	12	576.51	2.92	236.32	3
C25	0	2	222.24	3.46	98.14	1
Reference ligand	2	4	347.42	3.32	151.64	5

*Molecular weight (MW), number of hydrogen bond donors (HBD), number of hydrogen bond acceptors (HBA), number of rotatable bonds (RB), total polar surface area (t-PS), and log (octanol-water) partition coefficient (logP).

### 3.8 Molecular dynamics simulations

The binding of drugs to their targets has been extensively studied using molecular dynamics (MD) simulation. This involves defining the impact of specific mutations on the resistance profile of numerous drugs, accurately assessing the strength of the binding between a ligand and its target and examining the structure of macromolecules. Two substances (C3 and C8) that showed strong binding patterns against the HIV-RT and ZV-RdRP proteins, respectively, in this test were suggested for further MD simulation research.

#### 3.8.1 RMSD, RMSF, and RDF analysis for HIV-RT and ZV-RdRP complexes

**FIGURE 4:**
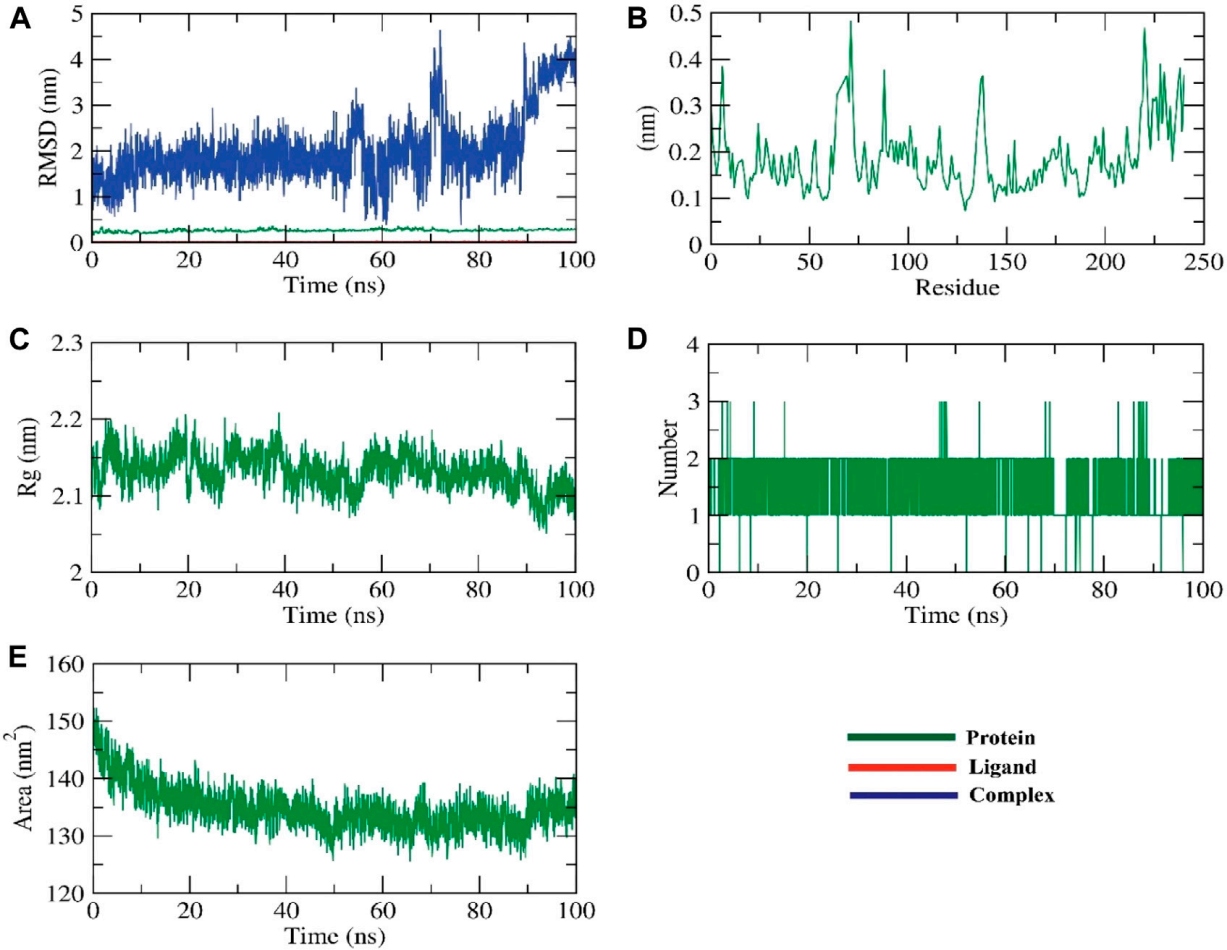
The MD simulations for the HIV-RT protein **(A)** RMSD analysis, **(B)** RMSF analysis, and **(C–E)** Radial distribution function (RDF).

**FIGURE 5:**
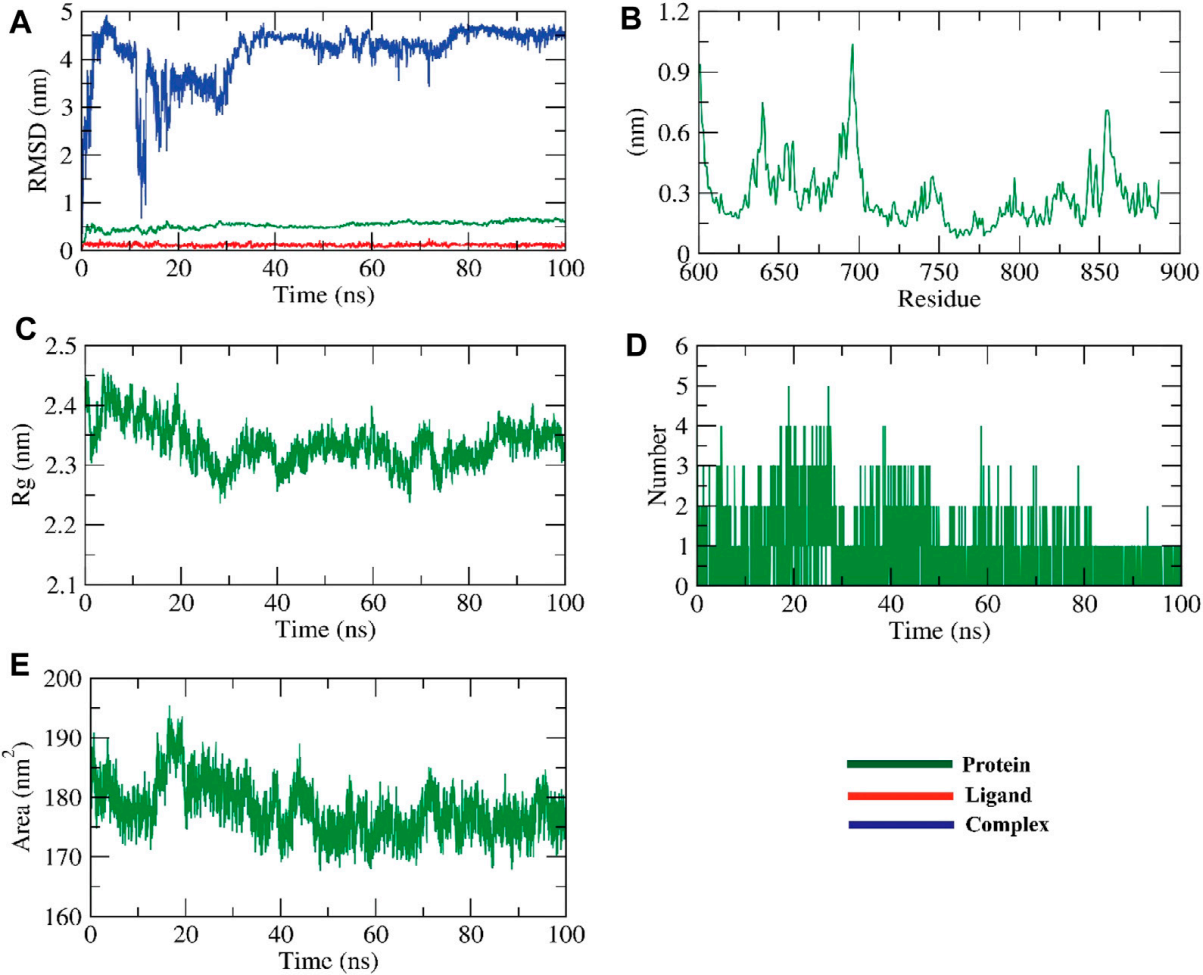
The MD simulations for the ZV-RdRPprotein **(A)** RMSD analysis, **(B)** RMSF analysis, and **(C–E)** Radial distribution function (RDF).

RMSD simulations were used to determine the relative stability of the protein-ligand complex’s backbone atoms by revealing their dynamic atom movements and conformational changes between the apo and ligand-bonded states. Lower RMSD values and indiscernible fluctuations during the simulation both indicate increased stability of the protein and ligand. The complex was stable for about 90 ns before showing signs of variation. To learn more about the regions of proteins that change during the simulation, we computed the flexibility of each residue in terms of RMSF. It makes sense that HIV-RT remains rigid in the region between 627 and 700 residues during ligand interaction but bends somewhat in this area after ZV-RdRP binding. The radius of gyration (Rg) was used to quantify the complex’s level of packing density. Reduced volatility during simulation is indicative of a system becoming more compact. It was discovered that the complex’s Rg was less than the initial phase’s Rg. Through the use of solvent accessible surface area (SASA), we were able to quantify the interactions between solvents and protein-ligand complexes that were simulated. The extent of conformational changes brought on by the interaction was evaluated by calculating the SASA of the complex. The protein’s SASA score and surface area were both smaller than they had been initially, which is an interesting finding. Stability of a protein-ligand complex is dependent on the formation of hydrogen bonds. Most protein conformations were shown to make up to two hydrogen bonds with the ligand (Figure 4; Figure 5).

#### 3.8.2 Binding free energy and contribution energy calculations using MM-PBSA approach for HIV-RT and ZV-RdRP complexes

##### 3.8.2.1 HIV-RT complexes

The dynamic movements of atoms and conformational variations of backbone atoms of the protein-ligand complex were calculated by RMSD to detect their stability upon apo and ligand bonded state. It is observed that the protein and ligand exhibit lower RMSD with no significant fluctuations throughout the simulation, indicating their more excellent stability. The complex was stable till −90 ns and fluctuated later. The flexibility of each residue was calculated in terms of RMSF to get a better insight into the region of proteins that fluctuate during the simulation. It can be understood that the binding of ligands does not make the protein flexible in any residue areas. The compactness of the complex was represented by the radius of gyration (Rg). The lower degree of fluctuation throughout the simulation period indicates the greater compactness of a system. The Rg of the complex was found to be lower than the starting period. Interaction between protein-ligand complexes and solvents was measured by solvent accessible surface area (SASA) over the simulation period. So, the SASA of the complex was calculated to analyze the extent of the conformational changes that occurred during the interaction. Interestingly, the protein reduced the surface area, showing a relatively lower SASA value than the starting period. Hydrogen bonding between a protein-ligand complex is essential to stabilize the structure. It was observed that the highest number of conformations of the protein formed up to two hydrogen bonds with the ligand, figure (Jin et al., 2019).

##### 3.8.2.2 MM-PBSA- HIV-RT complexes

**FIGURE 6:**
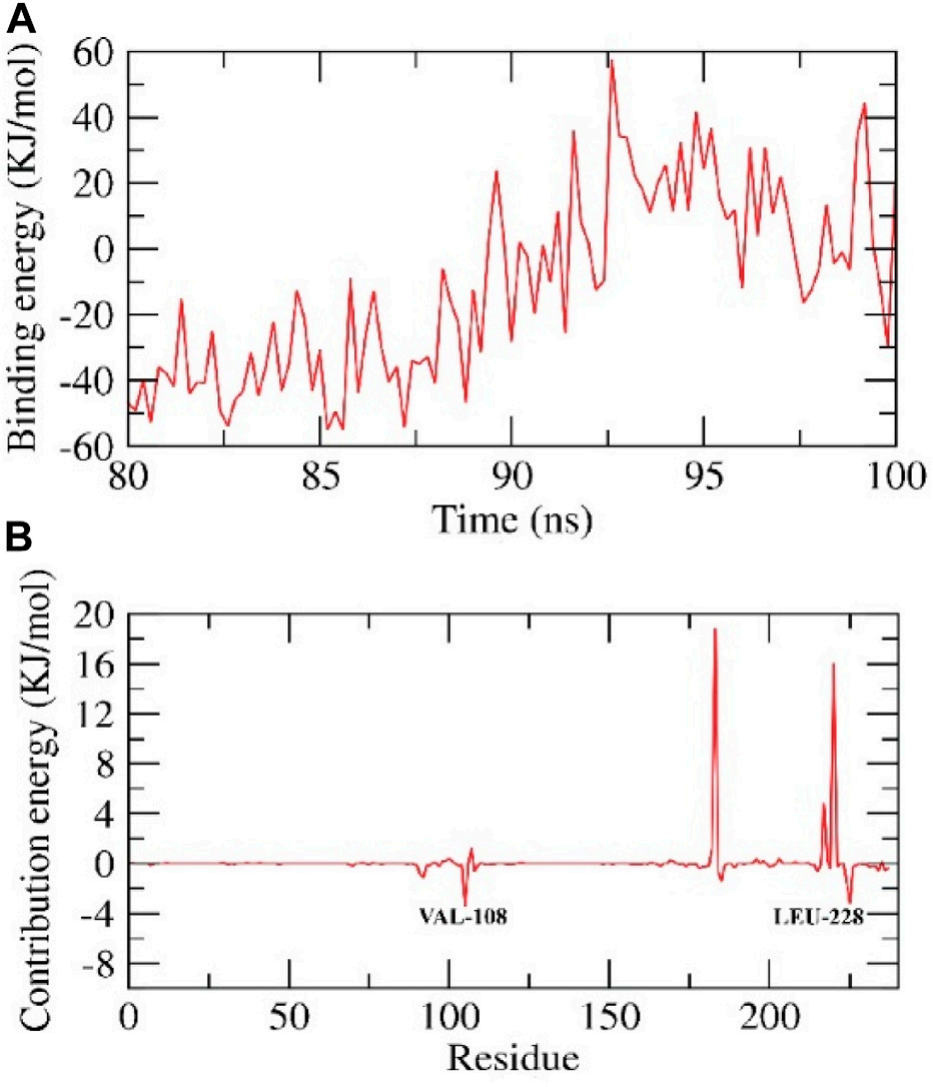
Contribution energy and the binding free energy for the HIV-RT complex. The binding free energy **(A)**, and contribution energy **(B)**, for the HIV-RT complex.

We calculated the binding free energy of the last 20 ns of the MD production run of the protein-ligand complex with an interval of 100 ps from MD trajectories using the MM-PBSA method. We also utilized the MmPbSaStat.py script that calculated the average free binding energy and its standard deviation/error from the output files obtained from g_mmpbsa. The ligand showed binding free energy of −11 kJ/mol with the protein. Further, we identified the contribution of each residue of the protein in terms of binding free energy to the interaction with the ligand. The contribution of each residue was calculated by decomposing the total binding free energy of the system into per residue contribution energy. This gave us an insight into the “crucial” residues that contribute favorably to the binding of this molecule to the protein. It was found that VAL-108 and LEU-228 residues of the protein contributed higher than −2 kJ/mol binding energy and, thereby, are hotspot residues in binding with the ligand, Figure 6.

##### 3.8.2.3 ZV-RdRP complexes

**FIGURE 7:**
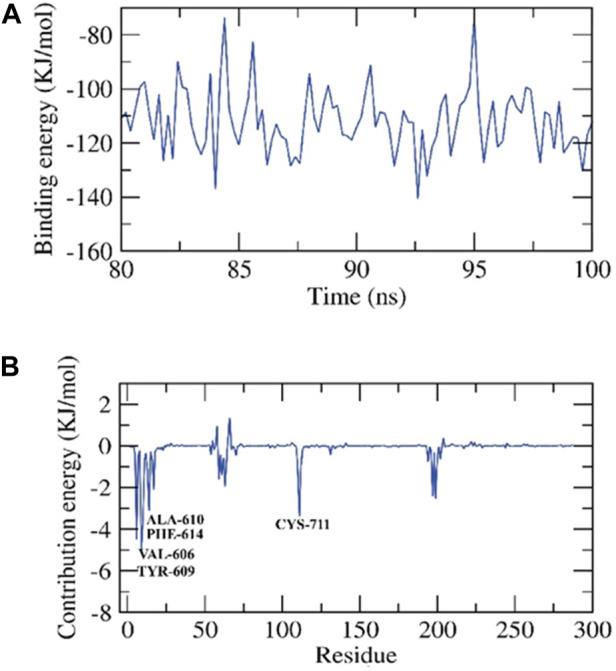
Contribution energy and the binding free energy for the ZV-RdRP complex. The binding free energy **(A)**, and contribution energy **(B)** for the ZV-RdRP complex.

RMSD was used to calculate the dynamic movements of atoms and conformational alterations of backbone atoms in the protein-ligand complex to assess their stability in the apo and ligand-bound states. The protein and ligand show consistent stability with slight variation during the simulation, as seen by their low RMSD values. The compound exhibited fluctuations until around 30 nanoseconds before reaching a stable state. RMSF was used to determine the flexibility of each residue to gain a deeper understanding of the protein regions that exhibit fluctuations during the simulation. The binding of ligands induces flexibility in the protein within the 625–700 residue regions. The complex’s compactness was shown by the radius of gyration (Rg). Less variation over the simulation time suggests a more condensed system. The radius of the gyration of the complex was smaller than that of the first period. The interaction between protein-ligand complexes and solvents was quantified using solvent accessible surface area (SASA) throughout the simulation. The SASA of the complex was determined to assess the degree of conformational changes that occurred during the contact. The protein exhibited a decrease in surface area, with a lower SASA value compared to the initial phase. Hydrogen bonding in a protein-ligand complex is crucial for stabilizing the structure. The protein exhibited the most conformations, creating a maximum of two hydrogen bonds with the ligand, Figure 7.

##### 3.8.2.4 MM-PBSA- ZV-RdRP complexes

We determined the binding free energy for the final 20 nanoseconds of the molecular dynamics production run of the protein-ligand complex. The calculations were based on MD trajectories and performed using the MM-PBSA technique with a 100-picosecond interval. We used the MmPbSaStat.py script to compute the mean free binding energy and its standard deviation/error from the output files generated by g_mmpbsa. The ligand exhibited a binding free energy of −111 kJ/mol with the protein. We determined the impact of each protein residue on the binding free energy about its interaction with the ligand. The individual contribution of each residue was determined by breaking down the overall binding free energy of the system into energy contributions per residue. This provided us with an understanding of the essential residues that positively impact the binding of this chemical to the protein. The protein’s VAL-606, TYR-609, ALA-610, PHE-614, and CYS-711 residues exhibited binding energies higher than −3 kJ/mol, making them hotspot residues in ligand binding, Figure 7.

## 4 Discussion

One of the most recently discovered microbially produced or synthesized biomolecules is called a biosurfactant. It is frequently used as a raw material in agricultural, waste management, or pharmaceutical industries for lubrication, wetting, foaming, emulsion formulations, or stabilising dispersions. (Muthusamy et al., 2008). According to certain definitions, the word “biosurfactant” refers to surface-acting substances that might enhance surface-surface interactions by generating micelles in the presence of natural sources, including plants, microorganisms, and animals (Al-Mokadem et al., 2022; Almuhayawi et al., 2023b). Additionally, biosurfactants have been used to lessen the surface tension at interfaces between solutions and surfaces, such as air/water or oil/water interfaces. (Rahman and Gakpe, 2008).

Many different types of microorganisms, such as fungi, bacteria, and yeasts, can produce biosurfactants (Maneerat and Phetrong, 2007). Some bacteria can thrive in conditions that would kill most other life forms. Among these extremophiles, halophiles are an essential microbial community due to their ability to thrive in high-salt environments; furthermore, many organisations value halophiles for the novel enzymes and other products they produce, which have many potential applications (Varjani and Upasani, 2017; Vaidya and Ganguli, 2019; Barbachano Torres et al., 2020; Sajid et al., 2020). Members of the genus Halobacterium are strictly aerobic and heterotrophic, and they can only be found in hypersaline environments like salt lakes and salterns. Phylogenetic analysis has led to the exclusion of many formerly included species of Halobacterium due to their distant relationships with other species in the genus (Abdelhadi et al., 2021). Halobacterium salinarum, described by (McGenity and Grant, 1995; Ventosa and Oren, 1996; Jones and Grant, 2000), describes Halobacterium noricense. Biosurfactant-producing bacteria are increasingly being studied. We’ve proven that highly halophilic bacteria are a promising place to look for biosurfactants due to the wide range of applications for these compounds. In this study, the method used to find halophilic bacterial strains that could make biosurfactants in very salty environments is described. The emulsification activity assay and the qualitative drop-collapse test are used to see how well a strain can make more biosurfactant. Phenotypic selection and partial sequencing of the 16S rRNA gene are also used in the identification process. *Bacillus* tequilensis ZSB10, a moderately halophilic bacterium, was found in a saltwater pond in the Tehuacan-Cuicatlan region of Mexico. Alpizar-Reyes et al. and Santos et al. (Hsueh et al., 2007; Wong et al., 2016) say that it made a new biosurfactant. According to (Santos et al., 2017; Alpizar-Reyes et al., 2022), the emulsification index (E24) for the halophilic bacteria under study in this study was 68%. Thus, emulsification activity is recognised as a very reliable and accurate approach to screening for the generation of biosurfactants (SOFY et al., 2022), especially when paired with the drop collapse test (Wani et al., 2018). Based on their molecular weight, biosurfactants can be classified as either “low” or “high.” Examples of high-molecular-weight biosurfactants include lipoproteins, polysaccharides, and lipopolysaccharides, while examples of low-molecular-weight biosurfactants include glycolipids, flavonoids, and lipopeptides (Sofy et al., 2020). Because halophilic bacteria from a wide variety of saltwater habitats have been shown to be bioactive against a wide variety of diseases, the pharmaceutical industry is devoting more attention to biomolecule applications. There is still a lot to learn about the bioactivities of halobacteria, halofungi, haloarchaea, and ha-lo-diatoms. The biosurfactant from *H. jilantaiense* strain JBS1 was first chemically characterised and found to have fatty acid derivatives and lipopeptide compounds. This is like the halophilic *Bacillus* sp. BS3 that was discovered in Kaniyakumari, India. It made a lipopeptide biosurfactant with chemicals such as 13-Docosenamide (Kuyukina et al., 2016). Lipopolysaccharide biosurfactants have recently caught the attention of scientists because they are eco-friendly, work well as pesticides, and fight fungi, viruses, bacteria, and fungal infections (Youssef et al., 2004; Floris et al., 2018). In addition, the assessment of cytotoxicity plays a crucial role in the characterization of newly developed chemicals or materials that are designed to interact with human biological systems *in vivo*. The initial stage of assessing cytotoxicity frequently involves evaluating the degree to which the molecule disrupts the erythrocyte membrane, leading to the release of cellular material (Mnif and Ghribi, 2016). The hemolysis assay employed in this study possesses several advantageous characteristics, including cost-effectiveness, accessibility, and ease of execution. The findings of this investigation align with the results reported by Zhang et al. (2014), suggesting that the biosurfactant exhibits a comparatively low level of toxicity, as the acceptable limit for hemolysis is below 5%. Surface-active compounds may not directly alter virus activity; nevertheless, credible findings (Whitehead et al., 2019) imply otherwise. The antibacterial, anti-biofilm, anti-inflammatory, and therapeutic activities of biosurfactants have been proven against a wide variety of pathogens (Venkatesan et al., 2014). Anti-adhesive, anti-viral, anti-cancer, anti-HIV, anti-inflammatory, immune-modulating, and antibacterial are just some of the impacts that surface-active compounds can have. In addition, molecular docking of the biosurfactant’s active components showed antiviral behavior against the zika virus and the human immunodeficiency virus. Like Zika, dengue virus (DENV) is a flavivirus (Culshaw et al., 2017; Shu et al., 2021; Elshazly et al., 2023; Hussein et al., 2023; Selim et al., 2023) that can cause serious illness or death. The use of broad-spectrum antiviral inhibitors might be extremely helpful in the treatment of ZIK virus and other flavivirus infections.

Conversely, Molecular Dynamics (MD) has served as a crucial computational instrument for examining various physical, chemical, and biological processes at the molecular scale. The findings of this analysis align with the study conducted by Fajardo-Rojas et al. (2020), it enables the estimation of the movement of atoms and particles over a period, given specific conditions, and the calculation of their statistical and thermodynamic characteristics by repeatedly solving the classical Newton’s equations of motion. In addition to the investigation of computational molecular docking, molecular dynamics simulations and binding energy techniques were employed to evaluate the molecule’s affinity for the HIV reverse transcriptase enzyme and ZIK virus. This is consistent with the findings of El Bakri et al. (2023), who demonstrated that the new quinoxaline derivative exhibits greater binding affinity and binding conformation stability with the HIV reverse transcriptase enzyme. These results suggest that the molecule holds promise as a potential candidate for further biological optimization. In addition, this work used the root mean square deviation (RMSD) to assess the consistency with the findings of Reva et al. (1998). The RMSD values calculated for all targeted proteins were found to be less than 20A, indicating a high-quality estimation. (Das et al., 2022), using Molecular Dynamics Simulation to assess the stability of the protein and the docked complex. Additionally, they investigated the impact of the interaction on HIV and Zika virus proteins. So, our present findings on Insilco are the basis of further studies to elucidate the mechanism of surfactant action. Additionally, this lipopeptide has important biotechnological and pharmacological potential as an anti-HIV and Zika virus compound that can be used to inactivate HIV and Zika viruses and make products safer for viruses.

## 5 Conclusion

We searched a natural chemical library and found three inhibitors that have not been previously reported to be effective against ZIKV and HIV infection. We have demonstrated that these all-natural chemicals are potent inhibitors of the spread of HIV and ZIKV. We have shown the synergistic benefits of biosurfactant natural products in warding off ZIKV and HIV infection by understanding their modes of action and potential targets. Our findings make it possible to do more research on these biosurfactant natural compounds and possibly use them as the main chemical in very powerful ZIKV and HIV inhibitors. In the future, we target studying the effect of biosurfactants isolated from microbes on HIV, Zika virus, and other viruses *in vitro* for application and ensuring *in silico* results. The biosurfactant currently under consideration exhibits a diverse array of potential applications due to its origin in halophilic *H. jilantaiense*. Using this substance in pharmacology could lead to its use as an ingredient in therapeutic formulations, which would improve drug transport and make it easier for the medicine to reach certain cell targets. Additional research is necessary to identify the potential applications of this biosurfactant across multiple fields.

## Data Availability

The original contributions presented in the study are included in the article/supplementary material, further inquiries can be directed to the corresponding authors.
