# Supplementary material for: Exploring biosurfactant from Halobacterium jilantaiense as drug against HIV and zika virus: fabrication, characterization, cytosafety property, molecular docking, and molecular dynamics simulation
Source: Front Bioeng Biotechnol. 2024 Mar 13;12:1348365. doi: 10.3389/fbioe.2024.1348365 (PMC10965788; doi:10.3389/fbioe.2024.1348365)
Supplement: Supplementary file 1 [file DataSheet1.pdf]

Exploring Biosurfactant from *Halobacterium jilantaiense* as Drug Against HIV and Zika virus: Fabrication, Characterization, Cytosafety property, Molecular Docking, and Molecular Dynamics Simulation.

Mohammed S. Almuhayawi[1]*, Naglaa Elshafey[2], Nashwa Hagagy[3,4], Samy Selim[5], Soad K. Al Jaouni[6], Ahmed R. Sofy[7], Mennatalla Samy[8], Hattan S Gattan[9,10], Mohammed H Alruhaili[1,10], Mohanned Talal Alharbi[11], Mohammed K Nagshabandi[11], Muyassar K Tarabulsi[11], Mohamed E. Elnosary[7]*

[1] Department of Clinical Microbiology and Immunology, Faculty of Medicine, King Abdulaziz University, Jeddah 21589, Saudi Arabia.

[2] Department of Botany and Microbiology, Faculty of Science, Arish University, Al-Arish 45511, Egypt.

[3] Department of Biology, College of Science & Arts at Khulis, University of Jeddah, Jeddah 21959, Saudi Arabia.

[4] Department of Botany and Microbiology, Faculty of Science, Suez Canal University, Ismailia 41522, Egypt

[5] Department of Clinical Laboratory Sciences, College of Applied Medical Sciences, Jouf University, Sakaka 72388, Saudi Arabia.

[6] Department of Hematology/Oncology, Yousef Abdulatif Jameel Scientific Chair of Prophetic Medicine Application, Faculty of Medicine, King Abdulaziz University, Jeddah 21589, Saudi Arabia.

[7] Department of Botany and Microbiology, Faculty of Science, Al-Azhar University, Nasr City, Cairo11884, Egypt

[8] Department of Communications and Computers Engineering, The Higher Institute of Engineering, El-Shorouk City 11837, Egypt

[9] Department of Medical Laboratory Technology, Faculty of Applied Medical Sciences, King Abdulaziz University, Jeddah, 21589, Saudi Arabia;

[10] Special Infectious Agents Unit, King Fahad Medical Research Center, King AbdulAziz University, Jeddah, 21589, Saudi Arabia;

[11] Department of Medical Microbiology and Parasitology, Faculty of Medicine, University of Jeddah, Jeddah, 23218, Saudi Arabia;

* Correspondence: msalmuhayawi@kau.edu.sa (Mohammed S. Almuhayawi), mohamed.elnosary@azhar.edu.eg (Mohamed E. Elnosary.)

Table S1. The HIV-RT and ZV-RdRP grid box and three-dimensional crystal structures in exquisite detail.

Protein	Code	Method	Resolution Å	Number of residues	Grid box dimension				
					X	Y	Z	spacing	npts
HIV-RT	5VZ6	X-RAY Diffraction	2.7	563	8.963	10.813	24.100	0.517	48 36 40
ZV-RdRP	5wz3		1.8	619	40.013	10.378	94.965	0.375	40 40 40

Table S2. The biosurfactant molecules by GC-MASS and ligand of HIV-RT as reference.

**Code**	**RT**	**Compound name**	**Molecular Formula**
C1	13.17	1,2,3-PROPANETRIOL,1,2-DIACETATE	C7H12O5
C2	13.17	Triacetin	C9H14O6
C3	18.82	1H-Cycloprop[e]azulen-7-ol,decahydro-1,1,7-trimethyl-4-methyle	C15H24O
C4	18.82	Ent-Spathulenol	C15H24O
C5	26.27	Pentadecanoic acid	C17H34O2
C6	26.27	Oxirane undecanoic acid	C19H36O3
C7	27.18	Hexadecanoic acid	C19H38O4
C8	29.15	Ethyl iso allocholate	C26H44O5
C9	29.15	Tetraneurin A	C15H20O5
C10	29.15	1-Heptatriacontanol	C37H76O
C11	29.55	Octadecadienoic acid	C18H34O2
C12	29.41	10-Octadecenoic acid	C19H36O2
C13	30.38	Ethyl linoleate	C20H36O2
C14	32.08	Isopropyl linoleate	C21H38O2
C15	32.08	Tricyclo[20.8.0.0(7,16)]triacontane,1(22),7(16)-diepoxy-	C30H52O2
C16	32.08	E,E,Z-1,3,12-Nonadecatriene-5,14-dIol	C19H34O2
C17	35.01	Stigmast-5-en-3-ol	C29H50O
C18	32.70	10,13-DIOXATRICYCLO [7.3.1.0(4,9)] TRIDECAN-5-OL-2-CARBOXYLIC ACID, 4-METHYL-11-(1-PROPENYL)-,METHYL ESTER	C17H26O5
C19	35.01	CIS-2-PHENYL-1,3-DIOXOLANE-4-METHYLOCTADEC-9, 12, 15-TRIENOATE	C28H40O4
C20	36.73	Diisooctyl phthalate	C24H38O4
C21	36.73	1,2-benzenedicarboxylic acid	C24H38O4
C22	36.73	Phthalic acid	C24H38O4
C23	38.49	Trilinolein	C57H98O6
C24	45.50	4H-1-BENZOPYRAN-4-ONE,2-(3,4-DIHYDROXYPHENYL)-6,8-DI-á-D-GLUCOPYRANOSYL-5,7-DIHYDROXY-	C27H30O16
C25	45.50	Flavone	C27H30O15
C26	45.50	Rhodopin	C40H58O
ref ligand		3-(pyrimidin-2-yl)-N-[3-(5,6,7,8-tetrahydronaphthalen-2-yl)-1H-pyrazol-5-yl] propenamide	C20 H21 N5 O

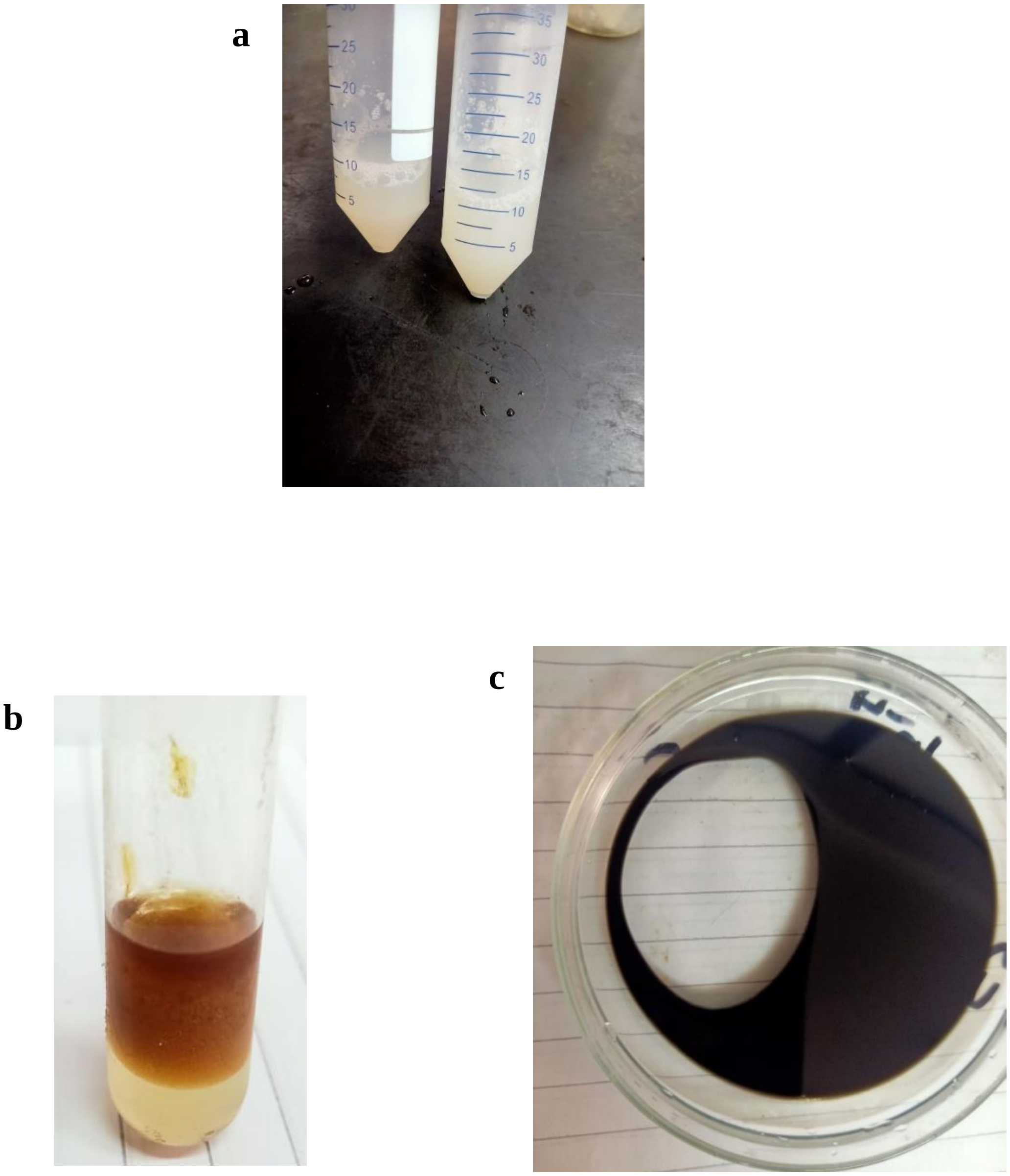

Fig. S1. Characteristics of biosurfactant produced by *Halobacterium jilantaiense*, (a) Biosurfactants in pellet form strain JBS1. (b) Showed the kerosene emulsification (68%) ;(c) showed Oil Displacement (2.9 cm).

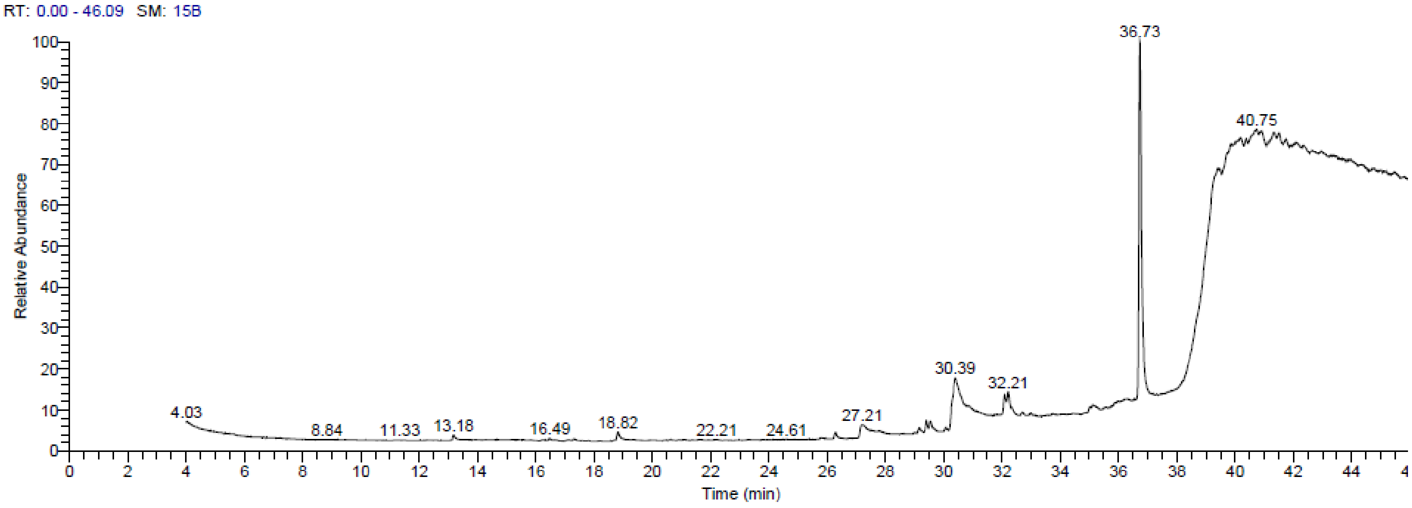

Fig. S2. GC-mass chromatogram of biosurfactant from promising *Halobacterium jilantaiense* strain JBS1

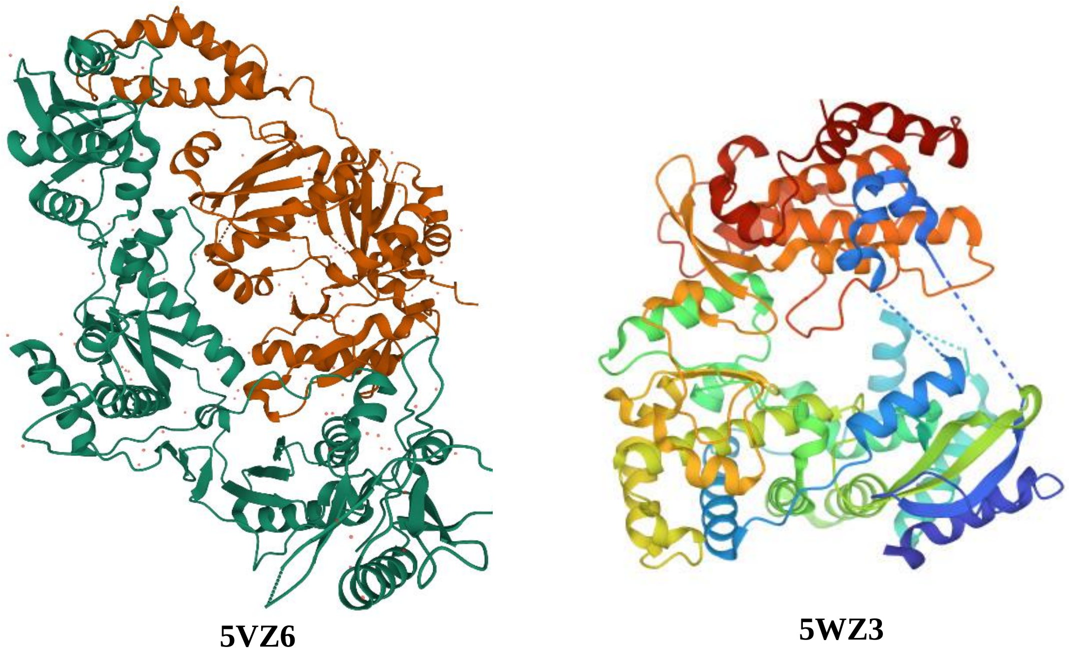

Fig. 4. 3-Dimensional structures of HIV (5VZ6) and Zika virus (5WZ3) target proteins.

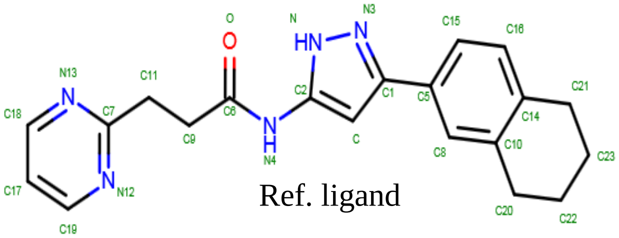

Ref. ligand

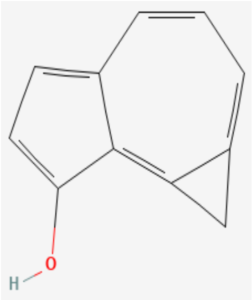

Docking interactions of C24

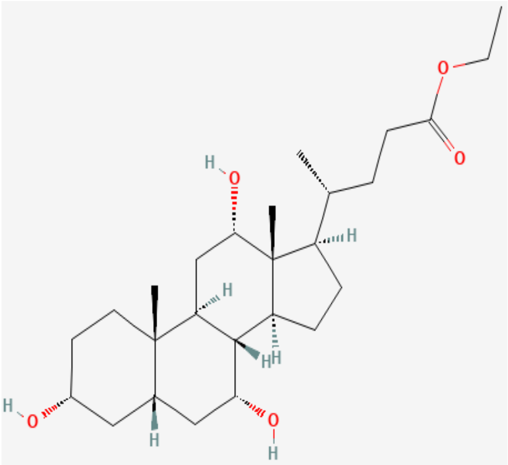

C8 ligand

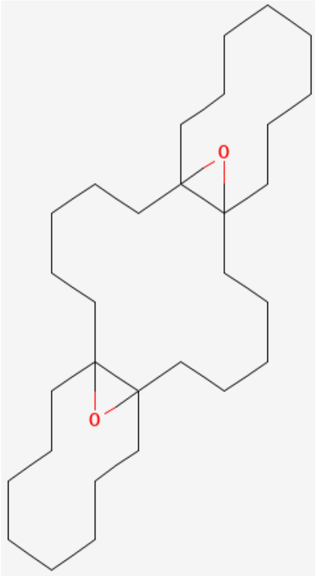

C15 ligand

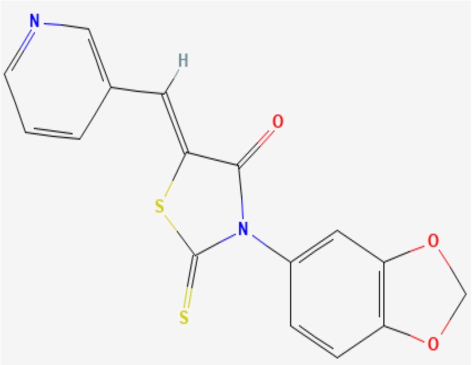

C24 ligand

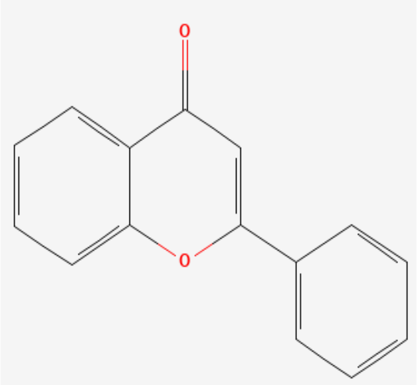

C25 ligand

Fig. S4. 2 D of top-ranked biosurfactant molecules.
